# Association Between Disturbed Sleep and Depression in Children and Youths: A Systematic Review and Meta-analysis of Cohort Studies

**DOI:** 10.1001/jamanetworkopen.2021.2373

**Published:** 2021-03-22

**Authors:** Cecilia Marino, Brendan Andrade, Susan C. Campisi, Marcus Wong, Haoyu Zhao, Xin Jing, Madison Aitken, Sarah Bonato, John Haltigan, Wei Wang, Peter Szatmari

**Affiliations:** 1 Centre for Addiction and Mental Health, Toronto, Ontario, Canada; 2 Department of Psychiatry, Temerty Faculty of Medicine, University of Toronto, Toronto, Ontario, Canada; 3 Department of Psychiatry, Hospital for Sick Children, Toronto, Ontario, Canada; 4 University of South Florida College of Public Health, Tampa

## Abstract

**Importance:**

Disturbed sleep represents a potentially modifiable risk factor for depression in children and youths that can be targeted in prevention programs.

**Objective:**

To evaluate the association between disturbed sleep and depression in children and youths using meta-analytic methods.

**Data Sources:**

Embase, MEDLINE, PsycINFO, Scopus, Web of Science, and ProQuest Dissertations & Theses Global were searched for articles published from 1980 to August 2019.

**Study Selection:**

Prospective cohort studies reporting estimates, adjusted for baseline depression, of the association between disturbed sleep and depression in 5- to 24-year-old participants from community and clinical-based samples with any comorbid diagnosis. Case series and reports, systematic reviews, meta-analyses, and treatment, theoretical, and position studies were excluded. A total of 8700 studies met the selection criteria. This study adhered to the guidelines of the Preferred Reporting Items for Systematic Reviews (PRISMA) and Meta-analyses and the Meta-analysis of Observational Studies in Epidemiology (MOOSE) statements.

**Data Extraction and Synthesis:**

Study screening and data extraction were conducted by 2 authors at all stages. To pool effect estimates, a fixed-effect model was used if *I*^2^ < 50%; otherwise, a random-effects model was used. The *I*^2^ statistic was used to assess heterogeneity. The risk of bias was assessed using the Research Triangle Institute Item Bank tool. Metaregression analyses were used to explore the heterogeneity associated with type of ascertainment, type of and assessment tool for disturbed sleep and depression, follow-up duration, disturbed sleep at follow-up, and age at baseline.

**Main Outcome and Measures:**

Disturbed sleep included sleep disturbances or insomnia. Depression included depressive disorders or dimensional constructs of depression. Covariates included age, sex, and sociodemographic variables.

**Results:**

A total of 22 studies (including 28 895 patients) were included in the study, of which 16 (including 27 073 patients) were included in the meta-analysis. The pooled β coefficient of the association between disturbed sleep and depression was 0.11 (95% CI, 0.06-0.15; *P* < .001; n = 14 067; *I*^2^ = 50.8%), and the pooled odds ratio of depression in those with vs without disturbed sleep was 1.50 (95% CI, 1.13-2.00; *P* = .005; n = 13 006; *I*^2^ = 87.7%). Metaregression and sensitivity analyses showed no evidence that pooled estimates differed across any covariate. Substantial publication bias was found.

**Conclusions and Relevance:**

This meta-analysis found a small but statistically significant effect size indicating an association between sleep disruption and depressive symptoms in children and youths. The high prevalence of disturbed sleep implies a large cohort of vulnerable children and youths who could develop depression. Disrupted sleep should be included in multifaceted prevention programs starting in childhood.

## Key Points

**Question:**

Is disturbed sleep associated with depression in children and youths?

**Findings:**

In this meta-analysis of 16 studies including 27 073 patients, pooled estimates supported the role of disturbed sleep as a risk factor for depression in children and youths. Effect size estimates were small but statistically significant.

**Meaning:**

This study suggests that disturbed sleep is a component of the multifaceted risk profile of depression and should be included in prevention programs as early as childhood.

## Teaser

This systematic review and meta-analysis evaluates the association between disturbed sleep and depression in children and youths.

## Introduction

Worldwide, depression is the third leading cause of disability, and its prevalence is increasing.^1^ Depression can start in childhood, and onset usually peaks at 20 years of age.^2^ Factors associated with first-onset depression in young adulthood include female sex, familial history of mood disorders, childhood sexual abuse, anxiety disorder, poor physical health, and subthreshold depressive symptoms, including disturbed sleep.^3^ Treatment of depression during adolescence has shown limited effectiveness.^4,5,6^ As a result, early identification and prevention may be a critical complementary tool to attenuate the risk trajectory of depression.

Emerging concepts for depression prevention point to multifaceted approaches to increase the level and duration of the intervention effects.^7^ Targeting modifiable risk factors is an additional, promising strategy for depression prevention. Multiple risk factors underpin an individual’s vulnerability to depression,^2^ and understanding the makeup of the risk profile is crucial for planning prevention programs. A meta-analysis of randomized clinical trials (RCTs) on depression prevention programs in 5- to 18-year-old participants found minimal impact that decayed over time, stressing the importance of developing innovative approaches.^8^

Disturbed sleep in adult and elderly individuals has been increasingly recognized as a risk factor for depression.^9,10,11,12,13,14^ Consequently, sleep has been the target of several selective and indicated depression prevention programs, with promising results.^15^

In children and youths, disturbed sleep and depression often co-occur in both clinical^16^ and community-based^17^ samples, with prevalence rates varying widely across studies.^18^ Disturbed sleep can be assessed by subjective measures, including child-, teacher-, or parent-reported questionnaires and interviews. The prevalence of disturbed sleep is 19.5% among children and is equally distributed among boys and girls; among youths, the prevalence rate is 17.4% and higher among girls (19.9%).^19^ There is evidence for a prospective association between disturbed sleep and depression in children and youths, whereas there is little support for the reverse association.^20,21^ Studies investigating the bidirectionality between the 2 constructs have provided mixed results,^22,23,24,25,26^ suggesting that a more complex cause may underlie this association. In a systematic review summarizing studies on the prospective association between insomnia and depression from 2014 to 2017, Pigeon et al^10^ found that, among 4 studies of children and youths, 3 found a significant association between insomnia and depression.^23,27,28^ Two meta-analyses provided quantitative data on the prospective association between disturbed sleep and depression in children and youths.^20,29^ The meta-analysis by Baglioni et al^29^ included 3 studies representing 7404 participants between 6 and 16 years of age and provided a pooled odds ratio (OR) of 2.0 (95% CI, 1.5-2.7) from community-based samples. The meta-analysis by Lovato et al^20^ included 7 studies representing 283 individuals with clinical depression aged 12 to 20 years and reported pooled effect sizes ranging from 0.43 to 0.58. The extent to which these pooled estimates differed across common moderators is currently unknown.

Whether treating sleep disturbances reduces depression in children and youths is still debated. A recent meta-analysis reported a pooled effect size in the small range based on 4 RCTs of youths with subthreshold mental health problems.^30^ Similar RCTs of children and in clinical populations are lacking, to our knowledge.

The primary aim of this study was to provide an updated systematic review and meta-analysis of prospective cohort studies that examined the association of disturbed sleep with the development of later depression among 5- to 24-year-old individuals. For a wider scope compared with previous reviews, we opted for broad definitions of disturbed sleep and depression. Based on prior systematic reviews,^20,29^ we expected to find that the pooled effect sizes were significant and in the small range.

## Methods

The Meta-analysis of Observational Studies in Epidemiology (MOOSE) reporting guideline^31^ was followed and fulfilled. This study also adhered to the Preferred Reporting Items for Systematic Reviews and Meta-analyses (PRISMA) guideline.

### Search Strategy, Study Selection, and Data Extraction

The search strategy is detailed in eMethods 1 in the Supplement. Title and abstract screening was performed by 2 authors (M.W. and X.J.) until a κ score^32^ of 0.8 was achieved, with the remainder being selected by 1 reviewer (M.W. or X.J.). Full-text screening was performed independently by 2 authors (M.W. and X.J.). Disagreements were solved by discussion and, if needed, referred to a third, senior-author reviewer (C.M.).

Studies were included if they were prospective, observational studies reporting quantitative estimates of the association between disturbed sleep and depression in participants belonging to either community or clinical-based samples with any comorbid psychiatric, neurologic, or medical diagnosis. Case series, case reports, systematic reviews, meta-analyses, and retrospective, cross-sectional, treatment, theoretical, and position studies were excluded.

Studies were included if participants’ mean age at baseline was between 5 and 24 years. This age range was chosen to cover the whole age range at risk for depression in children and youths and to include the period of peak incidence for depression at 20 years of age.^2,33^ In our review, we identified the following 3 age categories: childhood, between 5 and 9 years of age; adolescence, between 10 and 19 years of age; and young adulthood, between 20 and 24 years of age.^34^ Studies were included if both disturbed sleep and depression were measured at a mean age between 5 and 24 years and if the duration between baseline and follow-up was at least 1 month.

Definitions of disturbed sleep and depression are in eMethods 2 in the Supplement. Studies were included if they reported estimates adjusted for depression at baseline and at least 1 common moderating factor, such as age, sex, and socioeconomic status. To avoid duplicate publications, studies that shared samples, sample sizes, 1 or more authors, setting, numbers of participants at baseline and follow-up, date and duration of the study, and exposure and outcome were excluded. Data extraction is described in eMethods 3 in the Supplement.

### Risk-of-Bias Assessment

Risk of bias of included studies was appraised using the 13-item version of the Research Triangle Institute Item Bank tool,^35,36^ which is a development of the 29-item original questionnaire.^37^ The 13-item Research Triangle Institute Item Bank is a tool for observational studies assessing selection, confounding, performance, attrition, detection, and reporting biases, with high interrater reliability (75%). We dropped 2 items because they were not relevant to the body of literature of the included studies and added 1 question to further refine the attrition bias domain. eTable 1 in the Supplement reports the final included 12 questions. The risk-of-bias appraisal was performed by 2 authors (M.W. and X.J.) independently, with discrepancies being resolved by discussion and, if needed, by a third author (C.M.). Items were scored as high, low, unclear, or not applicable risk of bias. An overall risk-of-bias score was given to each study, based on the ratio between high and valid items (excluding not applicable items) and an arbitrary cutoff of 50%.^35^ The Covidence systematic review software was used for title and abstract and full-text screening, as well as extraction and quality assessment for included studies.

### Data Synthesis and Data Analysis

Data were synthesized by meta-analysis if a minimum of 2 studies were available. Studies were included in the meta-analysis if they reported effect size estimates, measures of precision, and *P* values. The *I*^2^ index was applied to quantify the heterogeneity among different studies, describing the percentage of variability in effect size estimates that is due to heterogeneity.^38^ *I*^2^ ≥ 50% means substantial heterogeneity. A fixed-effect model was used if *I*^2^ < 50%. A Dersimonian and Laird random-effects model was used when *I*^2^ ≥ 50%, based on the assumption that observed variance is due to heterogeneity among studies in populations and methods, rather than sampling error. Pooled estimates of ORs and β coefficients were obtained by the inverse variance–weighted method to account for the precision of the effect size. Odds ratios were log-transformed prior to meta-analysis to achieve approximate normality.

Pooled estimates were calculated for combined insomnia and sleep disturbances. When multiple follow-ups were available from 1 study, the longest follow-up was used in the meta-analysis; if follow-ups were of equal duration, we arbitrarily opted to use the earliest follow-up.

We fit meta-regressions to explore the heterogeneity associated with type of ascertainment, definition of and assessment tool for disturbed sleep and depression, follow-up duration, disturbed sleep at follow-up, and age at baseline.^13^ When 3 groups were compared, dummy variables were generated for comparison. A permutation test was implemented to derive *P* values for each subgroup analysis. Because previous meta-analyses^16,29^ did not perform metaregressions, no a priori hypotheses were formulated regarding the outcome.

Sensitivity analysis was performed by excluding studies with a high risk-of-bias score and studies with less than 200 participants, arbitrarily chosen as a cutoff to denote small sample sizes. Potential publication bias was assessed by visual inspection of the symmetry of funnel plots, fail-safe numbers,^39^ and Egger tests.^40^

Studies not included in the meta-analysis were synthesized based on Synthesis Without Meta-analysis (SWiM) guidelines (eMethods 4 in the Supplement).^41^ The Grading of Recommendations Assessment, Development and Evaluation (GRADE) was used to evaluate the quality of evidence of synthesis with and without meta-analysis separately.^42^ Meta-analysis, metaregression, and a test of heterogeneity were performed using STATA, version 14 (StataCorp). All *P* values were from 2-tailed tests and results were deemed statistically significant at *P* < .05.

## Results

### Search Results

**Figure 1.**
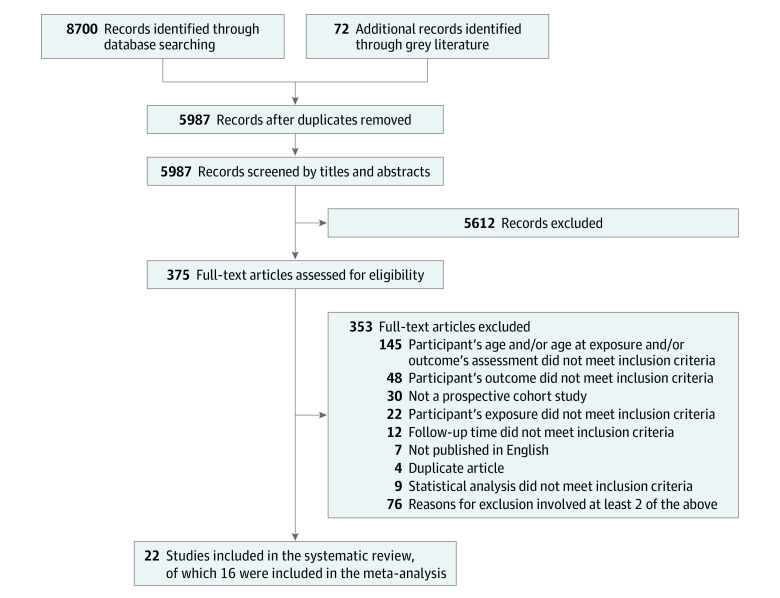
PRISMA Flow Diagram of Included Studies

After screening 8700 articles, 375 articles were selected for full-text review, and 22 studies were included.^22,25,27,43,44,45,46,47,48,49,50,51,52,53,54,55,56,57,58,59,60,61^ The PRISMA flowchart with studies included and excluded at each step, along with the rationale for the exclusion, is depicted in Figure 1.

### Studies Characteristics

The Table^22,25,27,43,44,45,46,47,48,49,50,51,52,53,54,55,56,57,58,59,60,61,62,63,64,65,66,67,68,69,70,71,72,73,74,75,76,77,78,79,80,81,82,83^ shows the characteristics of the included studies. eTable 2 and eResults in the Supplement show a summary of the included studies.

**Table.** Characteristics of Included Studies

Source	Sample	Follow-up		Sample size, No.	Age at baseline, mean (SD), y	Male sex, %	Factors associated with outcomes	Outcome	Confounders
		No.	Duration, mo						
**Dichotomous outcome**									
Fan et al,^48^ 2017 (China)	School-based cohort	1	12	1237	15 (1.3)	45.8	Sleep disturbance; PSQI^62^; self-report; sleep duration; broadly defined sleep problems; categorical	DSRS-C^63^; self-report; depression; categorical	Sex, age, residence location, only child, father’s educational level, earthquake exposure, and disturbed sleep at follow-up
Haraden et al,^60^ 2017 (US)	School-based cohort	1	12	360	15.0 (2.3) [range, 11-19]	43.5	Sleep disturbances; MESC^64^; self-report; chronotype; continuous	KSADS-PL^65^; interview; depression; categorical	Pubertal status
Kouros et al,^49^ 2016 (US)	School-based cohort; stratified on maternal depression	1	72	240	11.9 (0.6)	46	Sleep disturbances; CDRS-R^66^; interview; broadly defined sleep problems; categorical	LIFE^67^; interview; depression; categorical and continuous	Maternal depression, baseline sad mood, irritability, anhedonia, and sex
Luo et al,^51^ 2018 (China)	School-based cohort	1	24	246	17.7 (0.6)	49	Sleep disturbances; ESS^68^; self-report; daytime sleepiness; categorical	BDI^69^; self-report; depression; categorical	Age, sex, and disturbed sleep at follow-up
Luo et al,^52^ 2013 (China)	School-based cohort	1	12	2787	15.0 (1.5)	48.9	Insomnia; ISI^70^; self-report; continuous	BDI^69^; self-report; depression; categorical	Age, sex, and disturbed sleep at follow-up
Roane et al,^59^ 2008 (US)	School-based cohort	1	72	3582	15.8 (1.5)	47.6	Insomnia; single-item questionnaire: “How often did you have trouble falling asleep or staying asleep in the past 12 months?”; self-report; categorical	Single-item questionnaire: “Have you been previously diagnosed with depression?”; self-report; depression; categorical	Sex and existing mental disorder
Roberts et al,^53^ 2013 (US)	Community-based cohort	1	12	3134	Range, 11-17	51.1	Insomnia; 5-item questionnaire: “In the past 4 weeks, have you had (1) trouble falling asleep, (2) waking up in the middle of the night and finding it hard to go back to sleep, (3) waking up frequently but able to go back to sleep, (4) waking up very early, and (5) having nonrestorative sleep?”; self-report; categorical	DISC-IV^71^; interview; depression; categorical	Age, sex, and family income
Shanahan et al,^57^ 2014 (US)	Community-based cohort	NA	NA	1420	Range, 9-13	12	Sleep disturbance; CAPA^72^; interview; broadly defined sleep; continuous	CAPA^72^; interview; depression; categorical	Sex, race, age, pubertal status, and disturbed sleep at follow-up
Buysse et al,^44^ 2008 (Switzerland)	Community-based cohort; stratified on high risk for psychiatric syndromes	1	24	591	Male mean, 19; and female mean, 20	49.4	Insomnia; SPIKE^73^; interview; categorical	SPIKE^73^; interview; depression; categorical	Disturbed sleep at follow-up
Johnson et al,^46^ 2000 (US)	Community-based cohort; stratified on low birth weight and low SES	1	60	686	6	48.3	Sleep disturbance; single-item questionnaire: “Trouble sleeping during the past 6 mo”; self-report; broadly defined sleep problems	CBCL, TRF^74^; self-report; depression with anxiety; categorical	Mother’s history of depression, low birth weight, site (suburban vs urban), and sex
**Continuous outcome**									
Becker et al,^43^ 2015 (US)	Clinical-based cohort, ADHD	1	12	81	12.2 (1.0) [range, 10-14]	75	Sleep disturbance; CBCL sleep items^74^; self-report; broadly defined sleep problems; continuous	RADS-2^75^; self-report; depression; continuous	Age, sex, race, comorbid ODD, CD, anxiety disorder, and ADHD
Doane et al,^22^ 2015 (US)	School-based cohort	3	5, 4, 9	82	18.1 (0.41)	24	Sleep disturbance; Actiwatch (Phillips Respironics Inc); objective; sleep onset latency, sleep duration; PSQI^62^; self-report; broadly defined sleep problems; continuous	CES-D^76^; self-report; depression; continuous	Sex, race/ethnicity, parental educational level, living at home, and disturbed sleep at follow-up
Gregory et al,^45^ 2009 (UK)	Clinical-based, twin cohort	1	24	250	8	43	Sleep disturbance; CSHQ^77^; self-report; broadly defined sleep problems; continuous	CDI^78^; self-report; depression; continuous	Age, sex, and disturbed sleep at follow-up
Chang et al,^47^ 2017 (China)	School-based cohort	1	12	1893	Male, 14.7 (0.5); and female, 14.7 (0.5)	51.2	Sleep disturbance; PSQI^62^; self-report; broadly defined sleep problems; continuous	CES-D^76^; self-report; depression; continuous	Sex, age, family structure, family economic stress, stressful life events, pubertal development, prior peer victimization, and prior sleep problems
El-Sheikh et al,^61^ 2010 (US)	School-based cohort	1	24	176	8.7 (0.4)	44	Sleep disturbance; CSHQ^77^; parent-report; SHS^79^; self-report; broadly defined sleep problems; daytime sleepiness; continuous; Actigraph (Ambulatory Monitoring Inc); sleep duration; objective; continuous	PIC^80^; parent-report; CDI^78^; self-report; depression with anxiety; continuous	Sex, SES, and ethnicity
Alvaro et al,^27^ 2017 (Australia)	School-based cohort	1	6	255	15.0 (1.3)	NA	Insomnia; ISI^70^; self-report; continuous	RCADS^81^; self-report; depression; continuous	Age, sex, and chronotype
Ksinan Jiskrova et al,^50^ 2019 (Czech Republic)	General population, birth cohort	2	48, 36	3485	7	51.2	Sleep disturbances; 7-item questionnaire^82^; parent-report; sleep duration; chronotype; broadly defined sleep problems; continuous	SDQ^83^; parent-report; depression with anxiety; continuous	Sex, family structure, SES, maternal internalizing problems, and disturbed sleep at follow-up
Schneiderman et al,^54^ 2018 (US)	Case-control sample, maltreatment vs no maltreatment	1	54	Case, 247; control, 138	13.7 (1.4)	48.3	Sleep disturbance; PSQI^62^; self-report; broadly defined sleep problems; sleep duration; continuous	CDI^78^; self-report; depression; continuous	Age, sex, and disturbed sleep at follow-up
Wong et al,^55^ 2012 (US)	Community-based cohort	2	12, 12	6504	16.0 (1.8)	NA	Sleep disturbance; single-item questionnaire: “Please tell me how often you have had each of the following conditions in the past 12 months? – trouble falling asleep or staying asleep”; self-report; broadly defined sleep problems; continuous	CES-D^76^; self-report; depression; continuous	Age, sex, race, school grade, parental poverty, chronic health problems (allergies, asthma, migraines, diabetes, or obesity), alcohol and drug use disorders, and disturbed sleep at follow-up
Urrila et al,^56^ 2014 (Finland)	Clinical-based cohort, depression	1	13	142	16.5 (1.6) [range, 13-19]	17.5	Insomnia; KSADS-PL^65^; interview; continuous	BDI^69^; self-report; depression; continuous	Age, sex, within-individual correlation, and disturbed sleep at follow-up
Mulraney et al,^25^ 2016 (Australia)	Case-control sample, ADHD vs control	3	6, 6, 12	270	10.1 (1.9)	85.9	Sleep disturbance; CSHQ^77^; self-report; broadly defined sleep problems; continuous	SDQ^83^; parent-report; depression; continuous	Age, sex, ADHD symptom severity, primary caregiver educational level, and disturbed sleep at follow-up
Reynolds et al,^58^ 2016 (US)	Community-based cohort	1	72	1089	9	51.7	Sleep disturbance; CSHQ^77^; parent-report; broadly defined sleep problems; wake after sleep onset; continuous	YSR^74^; self-report; depression with anxiety; continuous	Total family income, marital status, and sex

Abbreviations: ADHD, attention-deficit/hyperactivity disorder; BDI, Beck Depression Inventory; CAPA, Child and Adolescent Psychiatric Assessment; CBCL, Child Behavior Checklist; CD, conduct disorder; CDI, Children’s Depression Inventory; CDRS-R, Children’s Depression Rating Scale–Revised; CES-D, Center for Epidemiological Studies Depression Scale; CSHQ, Children’s Sleep Habits Questionnaire; DISC-IV, Diagnostic Interview Schedule for Children Version IV; DSRS-C, Depression Self-Rating Scale for Children; ESS, Epworth Sleepiness Scale; ISI, Insomnia Severity Index; KSADS-PL, Schedule for Affective Disorders and Schizophrenia for School-Age Children–Present and Lifetime Version; LIFE, Longitudinal Interval Follow-up Evaluation; MESC, Morningness–Eveningness Scale for Children; NA, not available; ODD, oppositional defiant disorder; PIC, Personality Inventory for Children; PSQI, Pittsburgh Sleep Quality Index; RADS-2, Reynolds Adolescent Depression Scale–second edition; RCADS, Revised Child Anxiety and Depression Scale; SDQ, Strengths and Difficulties Questionnaire; SES, socioeconomic status; SHS, Sleep Habits Survey; SPIKE, Structured Psychopathological Interview and Rating of the Social Consequences of Psychological Disturbances for Epidemiology; TRF, Teacher Report Form; YSR, Youth Self-Report.

### Risk-of-Bias Assessment

**Figure 2.**
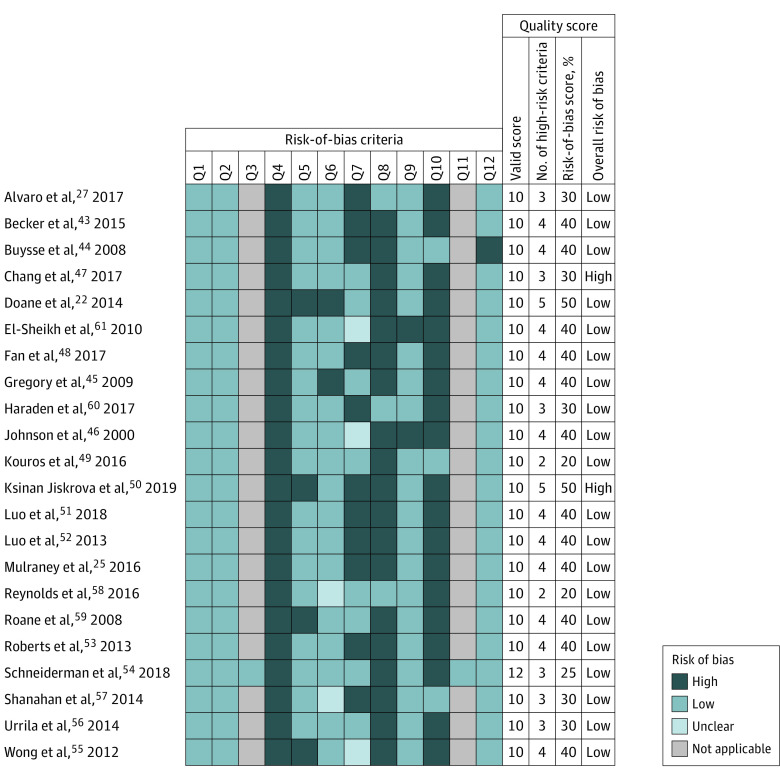
Risk-of-Bias Assessment of the Included Studies Based on the Research Triangle Institute Item Bank, by Heat Map The overall risk-of-bias score was based on the ratio between the number of high and valid items (excluding not applicable items) times 100; scores of 50 or more identified high risk of bias.

Figure 2^22,25,27,43,44,45,46,47,48,49,50,51,52,53,54,55,56,57,58,59,60,61^ reports the results of the risk-of-bias assessment by a heat map. The mean overall risk of bias was 36.1% (7.95 of 22), where lower scores indicated lower risk of bias. The mean number of high risk-of-bias responses was 3.64. Two studies^22,50^ were rated as having a high overall risk of bias (ie, overall risk-of-bias scoring ≥50%, which corresponded to 5 high risk-of-bias responses).^35,37^

### Synthesis With Meta-analysis

Sixteen studies presented sufficient data to allow synthesis via meta-analysis.^25,27,43,45,47,48,49,50,51,52,53,55,57,58,59,60^ Subgroup meta-analysis based on sex was precluded owing to insufficient data.

### Pooled Estimates of β Coefficients

**Figure 3.**
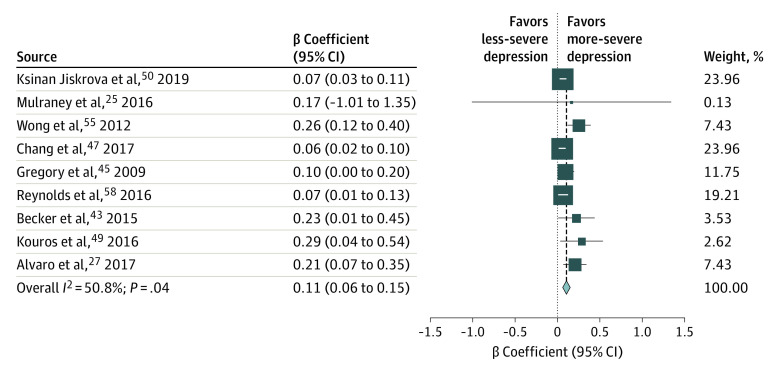
Forest Plot for Standardized β Coefficient Weights are from random-effects analysis.

Complete data on β coefficients were available from 1 study on insomnia and depression^27^ and from 8 studies on sleep disturbances and depression^25,43,45,47,49,50,55,58^ (eTable 3 in the Supplement). The pooled β coefficient of the association between disturbed sleep and depression was 0.11 (95% CI, 0.06-0.15; *P* < .001; n = 14 067; *I*^2^ = 50.8%; *P* = .04) (Figure 3). Metaregressions showed no evidence that the pooled estimate differed significantly across any covariate (eTable 4 in the Supplement). In sensitivity analysis that excluded studies with a high risk-of-bias score and studies of less than 200 participants, the pooled estimate remained of comparable magnitude (0.08; 95% CI, 0.05-0.11; *P* < .001; *I*^2^ = 34.9%; *P* = .16) and heterogeneity (0.10; 95% CI, 0.06-0.14; *P* < .001; *I*^2^ = 51.3%; *P* = .045).

### Pooled Estimates of ORs

**Figure 4.**
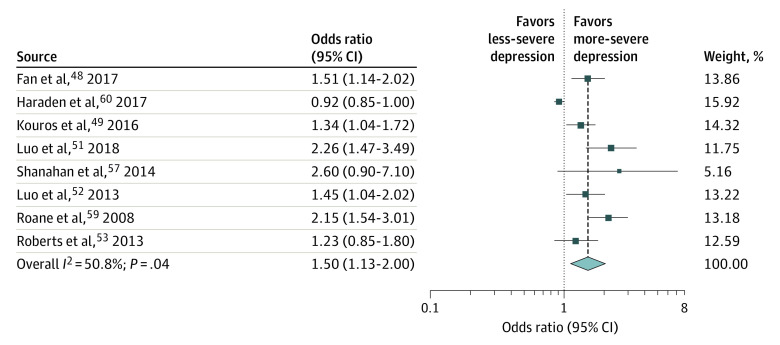
Forest Plot for Odds Ratio

Complete data on ORs were available from 3 studies on insomnia and depression^52,53,59^ and from 5 studies on sleep disturbances and depression^48,49,51,57,60^ (eTable 5 in the Supplement). In 3 studies,^48,51,52^ depression was based on cutoff scores applied to self-reported questionnaires (ie, 20 in the Beck Depression Inventory^69^ and 15 in the Depression Self-Rating Scale for Children^63^). Both cutoffs had been validated to identify depression with acceptable psychometric scores.^84,85^ The pooled OR estimate of depression in those with vs without disturbed sleep was 1.50 (95% CI, 1.13-2.00; *P* = .005; n = 13 006; *I*^2^ = 87.7%; *P* < .001) (Figure 4). Metaregressions showed no evidence that the pooled estimate differed significantly across any covariate (eTable 6 in the Supplement). All studies had low risk-of-bias scores and more than 200 participants; therefore, sensitivity analyses were not performed.

### Publication Bias

Visual inspection of the funnel plots identified substantial asymmetry for both pooled estimates (eFigures 1 and 2 in the Supplement). The Egger test identified substantial publication biases. We performed a leave-one-out sensitivity analysis by iteratively removing 1 study at a time and recalculating the pooled OR and β. Point estimates were within the 95% CI of the complete analysis (eFigures 3 and 4 in the Supplement). For both pooled estimates, the fail-safe number was larger than the number of studies (ie, 170 for the β coefficient and 92 for the OR coefficient).

### Quality of Evidence

According to GRADE,^42^ the overall body of evidence was rated as very low (eTable 8 in the Supplement), which means that the confidence in the effect size estimates is very limited.

### Synthesis of Studies Without Meta-analysis

For 6 studies,^22,44,46,54,56,61^ inclusion in the meta-analysis was precluded owing to effect size estimates being incomplete (eTable 7 in the Supplement). For the dichotomous outcome, disturbed sleep was significantly associated with an increase of depression in 1 study^44^ and not significantly associated in another.^46^ For the continuous outcome, disturbed sleep was significantly associated with an increase of depression in 1 study^56^ and was significantly associated with an increase of depression for the female subgroup only in an another study.^54^ Finally, 3 studies reported that findings were nonsignificant, although the direction of the findings was not clear.^22,54,61^ According to GRADE,^42^ the overall body of evidence was rated very low, which means that confidence in the effect size estimates is very limited (eTable 9 in the Supplement). Based on studies that provided direction of associations, the overall pattern supported an association between disturbed sleep and increased depression.

## Discussion

The present study provides summarized quantitative evidence of the prospective association between disturbed sleep and depression in children and youths after controlling for baseline depression. In line with our expectation, we found that the residual association after controlling for baseline depression was significant and of small magnitude. In fact, it is known that depression results from the joint action of a wide range of risk factors of small effect,^86^ and disturbed sleep is increasingly supported as one of these risk factors. Different mechanisms have been proposed to explain the association between disturbed sleep and depression, including an activation of inflammatory pathways, altered neuroplasticity and learning, and a disruption of circadian rhythm.^87^ Prevention of depression should target multicomponent risk profiles, possibly including sleep, and synthesis data are needed for the selection of the modifiable risk factors to target and the prioritization and timing of the prevention plan.^7^

Compared with previous meta-analyses on the association between disturbed sleep and depression in children and youths,^20,29^ our study is substantiated by a larger number of studies and broader inclusion criteria, which makes the results from this systematic review updated, more robust, and comprehensive. Furthermore, given that we included only studies controlling for baseline depression, pooled estimates depict the association between baseline disturbed sleep and residual depression at follow-up.

The pooled β coefficient of the association between disturbed sleep and depression was 0.11 (95% CI, 0.06-0.15) and was based on 9 studies representing 14 067 participants between 5 and 19 years of age.^25,27,43,45,47,49,50,55,58^ Metaregressions showed no evidence that the pooled estimate differed across any covariate, supporting the applicability of the pooled estimate to a broad spectrum of settings.

Metaregression shows that the pooled β coefficient did not differ by age at baseline, which is in line with previous literature,^20,29^ and showed that children with disturbed sleep are equally as vulnerable to later depression as youths. This finding is of particular relevance because it informs the timing of prevention strategies. Sleep problems are often underdiagnosed in childhood^88^ and are rarely treated,^89^ which may generate a large cohort of individuals vulnerable to depression.

The meta-analytic OR of the association between disturbed sleep and depression was 1.50 (95% CI, 1.13-2.00), in line with the pooled OR by Baglioni et al^29^ of 2.0 (95% CI, 1.5-2.7). The meta-analytic OR in our study was based on 8 studies representing 15 438 participants between 10 and 19 years of age.^48,49,51,52,53,57,59,60^ Despite heterogeneity, pooled studies were homogeneous along most characteristics (ie, age at baseline [10-19 years], type of ascertainment [community-based samples], depression without anxiety, sample size [>200], and risk-of-bias score [low]). Furthermore, metaregressions showed no evidence that the pooled estimate differed across any covariate, indicating that heterogeneity, although present, remains largely unexplained and may be clinically unimportant. Metaregression showed that the pooled OR did not differ according to the tool used for the assessment of disturbed sleep (single-item questionnaire vs multi-item questionnaire vs interview), supporting single-item questionnaires as valuable alternatives to more costly and time-consuming tools.

Strategies to prevent depression could start as early as childhood and possibly include treating disturbed sleep. Even though disturbed sleep can be effectively treated,^90,91,92,93,94^ only 2 RCTs targeting improvement of sleep to prevent depression have been completed, and they included participants older than 12 years,^95,96^ suggesting that more RCTs are needed, especially for children.

### Limitations

The present meta-analysis has several limitations. First, broad definitions of disturbed sleep and depression may have been associated with the variance of estimates across studies and did not allow us to test whether subtypes of disturbed sleep and depression had different association patterns. Second, there was a possible publication bias in the results. However, the leave-one-out test indicated that results were not associated with any single study, and the fail-safe numbers indicated that findings were robust against publication bias. Third, we included only English-language literature. Fourth, we included studies that did and did not control for disturbed sleep at follow-up; therefore, we were not able to assess that the association under study was significant over and above within-time covariation with depression. However, metaregressions were not significant, suggesting that heterogeneity could not be associated with disturbed sleep at follow-up. Fifth, the pooled estimate of β coefficients in children was based on 3 studies, suggesting caution is warranted in the interpretation of this result. Sixth, our review was not limited to studies assessing both disturbed sleep and depression repeatedly at each time point and, therefore, could not clarify temporal precedence, directionality, or reciprocity of associations. Seventh, the pooled estimate of β coefficients included 3 clinical-based studies, which could have been associated with the effect size owing to a more restricted range of variability of measures compared with community-based studies. However, metaregressions showed no significant differences. Eighth, common to all meta-analyses, the pooled ORs were dependent on the scale of the disturbed sleep measures of each study. Ninth, we were not able to assess the associations separately by sex owing to insufficient data; therefore, we could not exclude the possibility that sex was a confounding variable.

## Conclusions

This systematic review and meta-analysis found that disturbed sleep was prospectively associated with depression and provides quantitative estimates of the pooled effect sizes among children and youths. Although the pooled effect sizes are small, the high prevalence of children and youths with disturbed sleep implies the existence of a large cohort of vulnerable individuals who could develop depression. Disturbed sleep could be one of the many risk factors to address in depression prevention programs. Randomized clinical trials targeting disturbed sleep as early as childhood are needed to inform the planning and evaluation of depression prevention programs. These findings must be considered in the context of the limitations and the overall very low quality of evidence of the included studies.

## Notes

Supplement. **eMethods 1.** Search Strategy **eMethods 2.** Study Selection **eMethods 3.** Data Extraction **eMethods 4.** Synthesis of Studies Without Meta-Analysis **eResults.** **eTable 1.** Included Questions for Risk of Bias Assessment From the Research Triangle Institute Item Bank (RTI-IB) Tool **eTable 2.** Summary of Included Studies (n=22) **eTable 3.** Included Studies with Beta Coefficients **eTable 4.** Meta-Regression Analysis of Pooled Beta Coefficients for the Association Between Disturbed Sleep and Depression **eTable 5.** Included Studies With Odds Ratio Values **eTable 6.** Meta-Regression Analysis of Pooled ORs for the Association Between Disturbed Sleep and Depression **eTable 7.** Synthesis of Studies Without Meta-Analysis by Applying a Simple Vote-Counting by Direction of Effects **eTable 8.** Grading of Recommendations Assessment, Development and Evaluation (GRADE) for the Studies Included in the Synthesis With Meta-Analysis **eTable 9.** Grading of Recommendations Assessment, Development and Evaluation (GRADE) for the Studies Included in the Synthesis Without Meta-Analysis **eFigure 1.** Funnel Plot for Standardized Beta **eFigure 2.** Funnel Plot for Odds Ratio **eFigure 3.** Leave-One-Out Forest Plot for Standardized Beta **eFigure 4.** Leave-One-Out Forest Plot for Odds Ratio **eReferences.**
