# Supplementary material for: Association Between Disturbed Sleep and Depression in Children and Youths: A Systematic Review and Meta-analysis of Cohort Studies
Source: JAMA Netw Open. 2021 Mar 22;4(3):e212373. doi: 10.1001/jamanetworkopen.2021.2373 (PMC7985724; doi:10.1001/jamanetworkopen.2021.2373)
Supplement: Supplement. — eMethods 1. Search Strategy eMethods 2. Study Selection eMethods 3. Data Extraction eMethods 4. Synthesis of Studies Without Meta-Analysis eResults. eTable 1. Included Questions for Risk of Bias Assessment From the Research Triangle Institute Item Bank (RTI-IB) Tool eTable 2. Summary of Included Studies (n=22) eTable 3. Included Studies with Beta Coefficients eTable 4. Meta-Regression Analysis of Pooled Beta Coefficients for the Association Between Disturbed Sleep and Depression eTable 5. Included Studies With Odds Ratio Values eTable 6. Meta-Regression Analysis of Pooled ORs for the Association Between Disturbed Sleep and Depression eTable 7. Synthesis of Studies Without Meta-Analysis by Applying a Simple Vote-Counting by Direction of Effects eTable 8. Grading of Recommendations Assessment, Development and Evaluation (GRADE) for the Studies Included in the Synthesis With Meta-Analysis eTable 9. Grading of Recommendations Assessment, Development and Evaluation (GRADE) for the Studies Included in the Synthesis Without Meta-Analysis eFigure 1. Funnel Plot for Standardized Beta eFigure 2. Funnel Plot for Odds Ratio eFigure 3. Leave-One-Out Forest Plot for Standardized Beta eFigure 4. Leave-One-Out Forest Plot for Odds Ratio eReferences. [file jamanetwopen-e212373-s001.pdf]

Supplementary Online Content

Marino C, Andrade B, Campisi SC, et al. Association between disturbed sleep and depression in children and youths: a systematic review and meta-analysis of cohort studies. *JAMA Netw Open.* 2021;4(3):e212373. doi:10.1001/jamanetworkopen.2021.2373

eMethods 1. Search Strategy
eMethods 2. Study Selection
eMethods 3. Data Extraction
eMethods 4. Synthesis of Studies Without Meta-Analysis
eResults.
eTable 1. Included Questions for Risk of Bias Assessment From the Research Triangle Institute Item Bank (RTI-IB) Tool
eTable 2. Summary of Included Studies (n=22)
eTable 3. Included Studies with Beta Coefficients
eTable 4. Meta-Regression Analysis of Pooled Beta Coefficients for the Association Between Disturbed Sleep and Depression
eTable 5. Included Studies With Odds Ratio Values
eTable 6. Meta-Regression Analysis of Pooled ORs for the Association Between Disturbed Sleep and Depression
eTable 7. Synthesis of Studies Without Meta-Analysis by Applying a Simple Vote-Counting by Direction of Effects
eTable 8. Grading of Recommendations Assessment, Development and Evaluation (GRADE) for the Studies Included in the Synthesis With Meta-Analysis
eTable 9. Grading of Recommendations Assessment, Development and Evaluation (GRADE) for the Studies Included in the Synthesis Without Meta-Analysis
eFigure 1. Funnel Plot for Standardized Beta
eFigure 2. Funnel Plot for Odds Ratio
eFigure 3. Leave-One-Out Forest Plot for Standardized Beta
eFigure 4. Leave-One-Out Forest Plot for Odds Ratio
eReferences.

This supplementary material has been provided by the authors to give readers additional information about their work.

© 2021 Marino C et al. *JAMA Network Open.*

eMethods 1. Search Strategy

The literature search was conducted by a health sciences librarian (S.B.) to identifiy all relevant literature on the longitudinal association between disturbed sleep and depression from 1980 (to focus on current diagnostic terminology from the DSM III) to August 2019. Database search strategy was limited to English-written literature and to observational studies using the search filter developed by the Scottish Intercollegiate Guidelines Network. Searches used a combination of MeSH and keywords terms relating to sleep disorders, depression/depressive disorder and children/adolescents/young adults. The Medline strategy was developed with input from the research team, piloted to ensure the sensitivity and efficacy of the final search strategy and reviewed by all of the participating authors. The vocabulary and syntax of the finalized Medline search was adapted for optimal searching of the following databases: EMBASE, MEDLINE, PsychINFO, Scopus and Web of Science. The grey literature was screened using the search checklist Grey Matters: “A Practical Search Tool for Evidence-Based Medicine” from the Canadian Agency for Drugs and Technologies in Health (CADTH), and by also searching other sources to identify relevant dissertations/PhD theses and conference publications, i.e. Proquest Dissertations and Theses, ProceedingsFirst, PapersFirst, Conference Abstracts from Embase, Google Scholar Search. The references of key selected articles was hand-searched for eligible studies. The results were compiled and duplicates removed using EndNote X7 (EndNote, Clarivate Analytics).

Search Strategy for Database Ovid MEDLINE: Epub Ahead of Print, In-Process & Other Non-Indexed Citations, Ovid MEDLINE® Daily and Ovid MEDLINE® <1980-Present>

1 exp Sleep Wake Disorders/
2 exp Sleep/
3 (apnea$ adj3 sleep$).mp.
4 (restless adj3 leg$ adj3 syndrome$).mp.
5 narcolep$.mp.
6 (night$ adj3 terror$).mp.
7 sleep$.mp.
8 dyssomni$.mp.
9 parasomni$.mp.
10 insomni$.mp.
11 exp Depressive Disorder/
12 exp Depression/
13 depress$.mp.
14 exp Child/
15 exp Adolescents/
16 exp Young Adult/
17 (child$ or juvenile$ or pubescen$ or prepubescen* or pre-pubescen* or teen$ or preteen$ or tween$ or youth$ or adoles$ or young$ adult$ or emerg$ adult$).mp.
18 exp Epidemiologic studies/
19 exp Case control studies/
20 exp Cohort studies/
21 case control.tw.
22 (cohort adj (study or studies)).tw.
23 cohort analy$.tw.
24 (follow up adj (study or studies)).tw.
25 (observational adj (study or studies)).tw.
26 longitudinal.tw.
27 retrospective.tw.
28 cross sectional.tw.
29 cross-sectional studies/
30 or/1-10
31 or/11-13
32 or/14-17
33 or/18-29

© 2021 Marino C et al. *JAMA Network Open.*

34	30 and 31 and 32 and 33
35	limit 34 to yr="1980 -Current" ’

© 2021 Marino C et al. *JAMA Network Open.*

eMethods 2. Study Selection

Defintion and assessment tools for disturbed sleep

Disturbed sleep included either insomnia or sleep disturbances. Sleep disturbances comprised patterns of increased/decreased/fragmented sleep duration, poor sleep quality, daytime sleepiness, nightmares/terror attacks, circadian disturbances, bedwetting, bruxism and/or night walking/talking. Disturbed sleep could be assessed by single- or multiple-item self-report questionnaires, or structured interview. To ensure the generalizability of findings, studies were excluded if sleep disturbances focused exclusively on one single feature of disturbed sleep, e.g. sleep duration, sleep onset latency, etc.

Defintion and assessment tools for depression

Depression, both as baseline and at follow-up, included any depressive disorder or dimensional constructs of depression, or item-level depression with or without anxiety. The assessment tools used at baseline and follow-up could be interviews or self-report questionnaires (single-item or multi-item), and the one used at baseline could be different or the same as that used at follow-up. Studies including participants with bipolar disorders were excluded given that disturbed sleep differ significantly in bipolar versus unipolar disorders.[1 2]

© 2021 Marino C et al. *JAMA Network Open.*

eMethods 3. Data Extraction

Data extraction was conducted by two authors independently (MW, XJ) and checked by a third author (CM) using a pre-defined data extraction form. Extracted variables pertained to the following domains: general study information, country, study characteristics (i.e. the year of publication, type of sample ascertainment, sample size, number and duration of follow up, characteristics of disturbed sleep and depression, covariates), and participant characteristics (i.e. age at baseline, sex). Estimates of association included OR or relative risk for a dichotomous outcome,[3] and standardized beta for continuous outcome,[4] each with their respective measurements of error and p-values. Discrepancies between the two authors were resolved by discussion, and if needed by a third reviewer. When relevant data were missing, corresponding authors were contacted to obtain the information.

© 2021 Marino C et al. *JAMA Network Open.*

eMethods 4. Synthesis of Studies Without Meta-Analysis

For internal consistency, the studies were grouped by type of outcome, i.e. dichotomous or continuous, and ordered according to the risk of bias, i.e. the findings of the study with high risk of bias were reported in the summary table at the end of its outcome section. When available, estimates of association included odds ratio or relative risk for the dichotomous outcome,[3] and standardized beta for the continuous outcome,[4] each with their respective measurements of error and p-values. When these estimates were not available, we extracted the direction and significance of effect, if available. For each study, the association between baseline disturbed sleep and depression at follow-up was categorized as 'decrease of depression' or 'increase of depression' or 'unreported finding' based on the direction of effect, and as 'significant' or 'not significant'. Furthermore, the sample size upon which the effect was based on, was categorized as 'small' or 'large' based on whether it was, respectively, smaller or larger/equal than 200 participants. Since not all studies reported effect estimates, measures of precision and/or precise p-values, to synthesise the effects, we applied a simple vote-counting by the direction of effect method.[5]

© 2021 Marino C et al. *JAMA Network Open.*

eResults

Study characteristics

Studies were published between 2000[6] and 2019[7] with the majority being published between 2010 and 2019 (n=18/22, 81.8%).[7-24] Twelve studies[6 7 12 15 19 21-27] had follow ups longer than 12 months versus 10 studies[8-11 13 14 16-18 20] with follow ups equal or shorter than 12 months. Follow up duration ranged from 4[11] to 72 months.[15 27] The majority of studies included participants of mean age at baseline of 10-19 years (n= 17/22, 77.27%),[8-11 13-18 20-25 27] 5 studies of 5-9 years.[6 7 12 19 26] The majority of studies recruited community-based samples (n= 15/22, 68.18%),[7 8 10-17 19 20 22 24 27] and 7 studies, clinical-based samples.[6 9 18 21 23 25 26] Clinical samples included participants at risk for psychiatric disorders,[25] with history of maltreatement,[21] or low birth weight[6], or affected by ADHD,[9 18] depression,[23] or anxiety.[26] Sixteen studies focused on sleep disturbances[6 7 9-16 18 19 21 22 24 26] and 6 on insomnia[8 17 20 23 25 27] The majority of studies reported depression without anxiety (n=17/22, 77.27%),[8-11 13-17 20-27] 5 studies reported depression with anxiety.[6 7 12 18 19] To measure the disturbed sleep, most studies employed multi-item questionnaires (n=13/22, 59.10%),[7-9 13 14 16-22 26] 4 used single-item questionnaires,[6 10 24 27] 3 used interviews,[15 23 25] and 2 used multiple tools.[11 12] To measure the outcome, most studies employed multi-item questionnaires (n= 15/22, 68.18%),[6-11 13 16-19 21 23 24 26] 4 used interviews,[15 20 22 25] 3 used both interviews and questionnaires[14] and 2 used multiple tools.[12 14] All studies recruited both male and female participants, with approximately equal balance between females and males. Most studies reported results without differentiating males and females,[6-20 22-27] and 1 study[21] reported results for males and females separately.

© 2021 Marino C et al. *JAMA Network Open.*

eTable 1. Included Questions for Risk of Bias Assessment From the Research Triangle Institute Item Bank (RTI-IB) Tool

**Domain of Bias**	**Question**[a]
Selection	Q1: Do the inclusion/exclusion criteria vary across individuals/groups of the study?
Selection, Confounding	Q2: Is the strategy for recruiting participants into the study the same across study groups/individuals of the study?
Selection, Confounding	Q3: Is the selection of the comparison group appropriate after taking into account feasibility and ethical considerations?
Detection	Q4: Is the outcome assessor not blinded to the exposure status of participants?
Detection, Confounding	Q5: Are valid and reliable measures, implemented consistently across all study participants used to assess inclusion/exclusion criteria, exposure, outcomes and confounding?
Attrition	Q6: Is the length of follow-up different across study groups/individuals?
Attrition, Detection	Q7: In cases of high loss to follow-up (or differential loss to follow-up), is the impact assessed (e.g., through sensitivity analysis or other adjustment method)?
Attrition, Detection	Q8: In cases of missing data, did author specify the rate, if missing data were missing at random, and how missing data were handled? [b]
Reporting	Q9: Are any important primary outcomes missing from the results?
Overall	Q10: Are results believable taking study limitations into consideration?
Confounding	Q11: Any attempt to balance the allocation between the groups of match groups (e.g. through stratification, matching, propensity scores)?
Confounding	Q12: Are important confounding variables not taken into account in the design and/or analysis (e.g., through matching, stratification, interaction terms, multivariate analysis, or other statistical adjustment such as instrumental variables)?

[a]Two items were dropped because they were not relevant to the body of literature of the included studies ("Are any important harms or adverse events that may be a consequence of the intervention/exposure missing from the results?" and "Does the study fail to account for important variations in the execution of the study from the proposed protocol?").

[b]One question was added to further refine the attrition bias domain ("In cases of missing data, did author specify the rate, if missing data were missing at random, and how missing data were handled?").

© 2021 Marino C et al. *JAMA Network Open.*

eTable 2. Summary of Included Studies (n=22)

**Categories**	**Number of Studies (%)**
Mean age at Baseline, Years	
5-9	5 (22.7)
10-19	17 (77.3)
Sample Size	
<200	3 (13.6)
>=200	19 (86.4)
Population Type	
Community or School Based	15 (68.2)
Clinical Based	7 (31.8)
Duration of Follow up, Months	
<=12	10 (45.5)
>12	12 (54.5)
Publication Year	
2000-2009	3 (13.6)
2010-2020	19 (86.4)
Predictor	
Sleep Disturbances	16 (72.7)
Insomnia	6 (27.3)
Outcome	
Depression	17 (77.3)
Depression with Anxiety	5 (22.7)
Predictor, Diagnostic Tool	
Interview	3 (13.6)
Self-report Questionnaire	13 (59.1)
Self-report One-item Questionnaire	4 (18.2)
Objective (EEG/Actigraph)	0 (0)
Multiple Tools	2 (9.1)
Outcome, Diagnostic Tool	
Interview	4 (18.2)
Self-report Questionnaire	15 (68.2)
Self-report One-item Questionnaire	1 (4.5)
Multiple tools	2 (9.1)
Analysis Controlling for Covariates	
Adjusted	8 (36.4)
Unadjusted	5 (22.7)
Both	9 (40.9)
Analysis by Sex	
Yes	1 (4.5)
No	21 (95.5)

© 2021 Marino C et al. *JAMA Network Open.*

eTable 3. Included Studies with Beta Coefficients

First author, year of publication	Predictor → outcome	Ascertainment	Risk of bias score: (threshold 50)	Sample size	Age at baseline	Follow up	Sleep tools	Depression tools	Standardized beta	SE	*P*-value
Alvaro et al,[8] 2017	Insomnia → depression	community-based	low	>=200	10 - 19	<=12 Months	ISI[28]	RCADS[29]	0.21	0.07-0.35	<0.05
Becker et al,[9] 2015	Sleep disturbances → depression	clinical-based	low	<200	10 - 19	<=12 Months	CBCL, sleep items[30]	RADS-2[31]	0.23	0.01-0.45	0.045
Gregory et al,[26] 2009	Sleep disturbances → depression	clinical-based	low	>=200	5 - 9	>12 Months	CSHQ[32]	CDI[33]	0.1	.01 - 0.18	<0.05
Chang et al,[10] 2017	Sleep disturbances → depression	community-based	low	>=200	10 - 19	<=12 Months	PSQI[34]	CES-D[35]	0.06	0.02	<0.01
Kouros et al,[15] 2016	Sleep disturbances → depression	community-based	low	>=200	10 - 19	>12 Months	CDRS-R[36]	LIFE[37]	0.29	0.13	<0.01
KsinanJiskrova et al,[7] 2019	Sleep disturbances → depression with anxiety	community-based	high	>=200	5 - 9	>12 Months	Ad hoc questionnaire	SDQ[38]	0.067	0.02	0.001
Mulraney et al,[18] 2016	Sleep disturbances → depression with anxiety	clinical-based	low	>=200	10 - 19	<=12 Months	CSHQ[32]	SDQ[38]	0.17	0.6	0.004

© 2021 Marino C et al. *JAMA Network Open.*

First author, year of publication	Predictor → outcome	Ascertainment	Risk of bias score: (threshold 50)	Sample size	Age at baseline	Follow up	Sleep tools	Depression tools	Standardized beta	SE	*P*-value
Reynolds et al,[19] 2016	Sleep disturbances → depression with anxiety	community-based	low	>=200	5 - 9	>12 Months	CSHQ[32]	YSR[30]	0.07	0.03	0.02
Wong et al,[24] 2012	Sleep disturbances → depression	community-based	high	>=200	10 - 19	<=12 Months	single-item[1]	CES-D[35]	0.26	0.07	<0.001

ISI= Insomnia Severity Index; RCADS= Revised Children's Anxiety and Depression Scale; CBCL= Child Behavior Checklist; RADS-2= Reynolds Adolescent Depression Scale, 2nd Edition; CSHQ= The Children's Sleep Habits Questionnaire; CDI= The Children's Depression Inventory; PSQI= The Pittsburgh Sleep Quality Index; CES-D= Center for Epidemiological Studies Depression; CDRS-R= Children's Depression Rating Scale – Revised; LIFE= Longitudinal Interval Follow-up Evaluation; SDQ= Strengths and Difficulties Questionnaire; YSR= Youth Self Report.

[1] "Please, tell me how often you have had each of the following conditions in the past 12 months? – trouble falling asleep or staying asleep"

© 2021 Marino C et al. *JAMA Network Open.*

eTable 4. Meta-Regression Analysis of Pooled Beta Coefficients for the Association Between Disturbed Sleep and Depression

	**Number of studies**	**Pooled beta**	**95% CI**	**I^2**	***P*-value heterogeneity**	***P*-value between groups**
Mean age at baseline (years)						
5-9	3	0.07	0.04, 0.10	0.0%	0.828	0.218
10-19	6	0.19	0.08, 0.30	66.8%	0.01	
Type of ascertainment						
Clinical-based	3	0.12	0.03, 0.21	0.0%	0.559	0.717
Community-based	6	0.11	0.06, 0.16	64.7%	0.015	
Disturbed sleep definition						
Insomnia	1	0.21	0.07, 0.35	/	/	0.227
Sleep disturbances	8	0.08	0.05, 0.10	44.9%	0.079	
Depression definition						
Depression	6	0.16	0.08, 0.25	66.8%	0.01	0.246
Depression with anxiety	3	0.07	0.04, 0.1	0.0%	0.982	
Disturbed sleep measure						
Self report, multi-item	7	0.12	0.07, 0.18	51.1%	0.056	0.17
Self report, single-item	1	0.06	0.02, 0.1	/	/	
Interview	1	0.29	0.04, 0.55	/	/	
Depression measure						
Self report, multi-item	8	0.08	0.06, 0.1	48.6%	0.058	0.333
Self report, single-item	0	/	/	/	/	
Interview	1	0.29	0.04, 0.55	/	/	
Follow up duration						
<= 12 y	5	0.17	0.06, 0.29	68.6%	0.013	0.31
> 12	4	0.08	0.04, 0.11	5.4%	0.366	
Sleep at follow up						
controlled	4	0.122	0.033,0.21	58.1%	0.067	0.975
Not controlled	5	0.112	0.047,0.177	55.6%	0.061	

© 2021 Marino C et al. *JAMA Network Open.*

eTable 5. Included Studies With Odds Ratio Values

**First author, year of publication**	**predictor → outcome**	**Population**	**risk of bias score (threshold of 50)**	**sample size:**	**Age at baseline**	**Follow up period**	**Sleep Tools**	**Depression Tools**	**OR**	**95% CI**	***P*-value**
Fan et al,[13] 2017	sleep disturbances→ depression	general	low	>= 200	10 - 19	< 12 Months	PSQI;[34]	DSRS-C[39]	1.51	1.14,2.02	<0.01
Haraden et al,[14] 2017	sleep disturbances→ depression	general	low	>= 200	10 - 19	< 12 Months	MESC[40]	KSADS-PL[41]	0.92	0.847, 0.996	0.03
Kouros et al,[15] 2016	sleep disturbances→ depression	general	low	>= 200	10 - 19	> = 12 Months	CDRS-R[36]	LIFE[37]	1.34	1.04,1.72	<0.01
Luo et al,[16] 2018	sleep disturbances→ depression	general	low	>= 200	10 - 19	< 12 Months	ESS[42]	BDI[43]	2.26	1.47,3.49	<0.01
Shanahan et al,[22] 2014	sleep disturbances→ depression	general	low	>= 200	10 - 19	> 12 Months	CAPA[44]	CAPA[44]	2.6	0.9,7.1	ns
Luo et al,[17] 2013	insomnia→ depression	general	low	>= 200	10 - 19	< 12 Months	ISI[28]	BDI[43]	1.45	1.04,2.02	<0.05
Roane et al,[27] 2008	insomnia→ depression	general	low	>= 200	10 - 19	> = 12 Months	single-item[1]	single-item[2]	2.15	1.54,3.01	<0.001
Roberts et al,[20] 2013	insomnia→ depression	general	low	>= 200	10 - 19	< 12 Months	Ad hoc questionnaire	DISC-IV[45]	1.23	.85,1.80	<0.05

PSQI= The Pittsburgh Sleep Quality Index; DSRS-C= Depression Self-Rating Scale for Children; MESC= Morningness–Eveningness Scale for Children; KSADS-PL= Schedule for Affective Disorders and Schizophrenia for School-Age Children-Present and Lifetime; CDRS-R= Children's Depression Rating Scale – Revised; LIFE= Longitudinal Interval Follow-up Evaluation; ESS= Epworth

© 2021 Marino C et al. *JAMA Network Open.*

sleepiness scale; BDI= Beck Depression Inventory; CAPA= The Child and Adolescent Psychiatric Assessment; ISI= Insomnia Severity Index; DISC-IV= Diagnostic Interview Schedule for Children, version IV;

[1] "How often did you have trouble falling asleep or staying asleep in the past 12 months?"

[2] "Have you been previously diagnosed with depression?"

© 2021 Marino C et al. *JAMA Network Open.*

eTable 6. Meta-Regression Analysis of Pooled ORs for the Association Between Disturbed Sleep and Depression

	**Number of studies**	**Pooled OR**	**95% CI**	**I^2**	***P*-value heterogeneity**	***P*-value between groups**
Mean age at baseline (years)						
5-9	0	/	/	/	/	/
10-19	8	/	/	/	/	
Type of ascertainment						
Clinical-based	0	/	/	/	/	/
Community-based	8	/	/	/	/	
Disturbed sleep definition						
Insomnia	3	1.58	1.14,2.18	61.6%	0.074	0.79
Sleep disturbances	5	1.46	1.01,2.1	88.3%	<0.01	
Depression definition						
Depression	8	/	/	/	/	/
Depression with anxiety	0	/	/	/	/	
Disturbed sleep measure						
Self report, multi-item	5	1.37	0.98,1.92	87.4%	<0.001	0.96
Self report, single-item	2	1.68	1.05,2.66	79.6%	0.027	
Interview	1	2.6	0.93, 7.3	/	/	
Depression measure						
Self report, multi-item	4	1.74	1.47,2.05	40.5%	0.169	0.09
Self report, single-item	0	\	\	\	\	
Interview	4	1.19	0.88,1.61	77.0%	0.005	
Follow up duration						
<= 12 y	5	1.37	0.98,1.92	87.4%	<0.001	0.47
> 12	2	1.68	1.05,2.66	79.6%	0.027	
Sleep at follow-up						
controlled	4	1.641	1.357,1.985	19.5%	0.292	0.312
Not controlled	4	1.32	0.906,1.922	90.2%	<0.001	

© 2021 Marino C et al. *JAMA Network Open.*

eTable 7. Synthesis of Studies Without Meta-Analysis by Applying a Simple Vote-Counting by Direction of Effects

**Study**	**Risk of bias**	**Sample size**	**Disturbed sleep --> depression**	**Effect[a]**	**Reason for exclusion from meta-analysis**
Dichotomous outcome					
Buysse et al,[25] 2008	low	591	Insomnia.<br>OR, p=significant	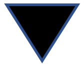	Insufficient data
Johnson et al,[6] 2000	low	686	Sleep disturbances. Findings available for subgroups only.<br>Suburban children, relative risk=2.22, 95% CI .53-9.23, p=not significant.<br>Urban children, relative risk= .92, 95% CI .20-4.18, p=not significant	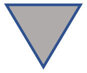	Subgroups only
Continuous outcome					
Urrila et al,[23] 2014	low	142	Insomnia.<br>Beta, p=significant	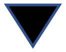	Insufficient data
El-Sheikh et al,[12] 2010	low	176	Sleep disturbances.<br>Beta: n.s.	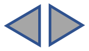	Insufficient data
Schneiderman et al,[21] 2018	low	247	Sleep disturbances. Findings available for subgroups only.<br>Female: beta=.157, p < .05; male: n.s.	females 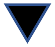<br>males 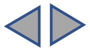	Subgroups only
Doane et al,[11] 2015	high	82	Sleep disturbances.<br>Beta: n.s.	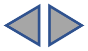	Insufficient data

[a]Sample size: small arrow (n<200); large arrow (n≥200). Effect direction: upward arrow (decrease in depression), downward arrow (increase in depression), sideways arrow (unreported findings). Statistical significance: black arrow (p<0.05); grey arrow (p≥0.05). Effect is adjusted by depression and confounders.

© 2021 Marino C et al. *JAMA Network Open.*

eTable 8. Grading of Recommendations Assessment, Development and Evaluation (GRADE) for the Studies Included in the Synthesis With Meta-Analysis

criteria	rating[g]	Reasons for downgrading/upgrading
Outcome: depression		
# studies<br># participants	16<br>29505	
Study design	Initial rate LOW	Non-randomized, follow-up, prospective. As per GRADE guidelines the initial rate is "low" due to concerns of confounding and selection bias resulting from the lack of randomization
Risk of Bias [a]	Not serious	1 study at high risk of bias
Inconsistency [b]	Serious	Significant heterogeneity, i.e. I^2 = 50.8%; p=0.039[i]; I^2 = 87.7%; p<0.001[l]; pooled beta coefficients vary from 0.06 to 0.29; pooled ORs vary from 0.92 to 2.6
Indirectness [c]	Not serious	2 studies used a single-item questionnaire to measure disturbed sleep
Imprecision [d]	Not serious	Only 2 studies had large 95% CIs
Publication bias[e]	Not serious	Publication bias was supported by funnel plots (eFigure 1 and eFigure2) and Egger's tests (p<0.05). However, the leave-one-out sensitivity analysis indicated that our results were not driven by any single study, and the 'fail safe number' test indicated that our findings were robust against publication bias
Upgrading factors[f]	Not affected	Both pooled estimates were of small size. The 'dose-response effect' and the 'plausible confounding affecting the effect' were not applicable.
Overall quality of evidence	⊕[h]	The initial rate 'low' was downgraded to 'very low' because of inconsistent effect estimates. The overall quality of evidence is 'very low', which means that the confidence in the effect estimates is very limited

[a] Risk of bias: based on the Research Triangle Institute Item Bank (RTI-IB) tool (see figure 2)
[b] Inconsistency: based on heterogeneity (I^2 statistic) and differing estimates of the effect across studies
[c] Indirectness: based on how well the evidence addresses the study hypothesis
[d] Imprecision: based on evaluation of width of confidence intervals
[e] Publication bias: as evaluated in this meta-analysis
[f] Upgrading factors: based on magnitude of effect, dose-response relationship and effect of confounding factors
[g] Not affected, Not serious (downgraded by 1), Very serious (downgraded by 2)
[h] Overall quality of evidence ranges ⊕⊕⊕⊕ High, ⊕⊕⊕ Moderate, ⊕⊕ Low, ⊕Very Low
[i] Pooled beta coefficient
[l] Pooled OR

© 2021 Marino C et al. *JAMA Network Open.*

eTable 9. Grading of Recommendations Assessment, Development and Evaluation (GRADE) for the Studies Included in the Synthesis Without Meta-Analysis

criteria	rating[g]	Reasons for downgrading/upgrading
Outcome: depression		
# studies<br># participants	6<br>1924	
Study design	Initial rate LOW	Non-randomized, follow-up, prospective. As per GRADE guidelines the initial rate is "low" due to concerns of confounding and selection bias resulting from the lack of randomization.
Risk of Bias [a]	Not serious	1 study at high risk of bias.
Inconsistency [b]	Not able to assess	Difficult to assess because the majority of studies did not report effect estimates and measures of precision. 4 studies reported a reduction of depression. For 2 studies, the direction of effect was not clear.
Indirectness [c]	Serious	1 study used a single-item questionnaire to measure disturbed sleep. 2 studies reported results for subgroups only. 3 studies did not report on direction of effects
Imprecision [d]	Not able to assess	Difficult to assess because the majority of studies did not report effect estimates and measures of precision.
Publication bias[e]	Not able to assess	Difficult to assess because the majority of studies did not report effect estimates.
Upgrading factors[f]	Not able to assess	Difficult to assess because not all studies reported effect estimates.
Overall quality of evidence	⨁[h]	The initial rate 'low' was downgraded to 'very low' because of indirectness. The overall quality of evidence is 'very low', which means that the confidence in the effect estimates is very limited

[a] Risk of bias: based on the Research Triangle Institute Item Bank (RTI-IB) tool (see figure 2)
[b] Inconsistency: based on heterogeneity (I^2 statistic) and differing estimates of the effect across studies
[c] Indirectness: based on how well the evidence addresses the study hypothesis
[d] Imprecision: based on evaluation of width of confidence intervals
[e] Publication bias: as evaluated by funnel plots, if possible
[f] Upgrading factors: based on magnitude of effect, dose-response relationship and effect of confounding factors
[g] Not affected, Not serious (downgraded by 1), Very serious (downgraded by 2)
[h] Overall quality of evidence ranges ⨁⨁⨁⨁ High, ⨁⨁⨁ Moderate, ⨁⨁ Low, ⨁Very Low

© 2021 Marino C et al. *JAMA Network Open.*

eFigure 1. Funnel Plot for Standardized Beta

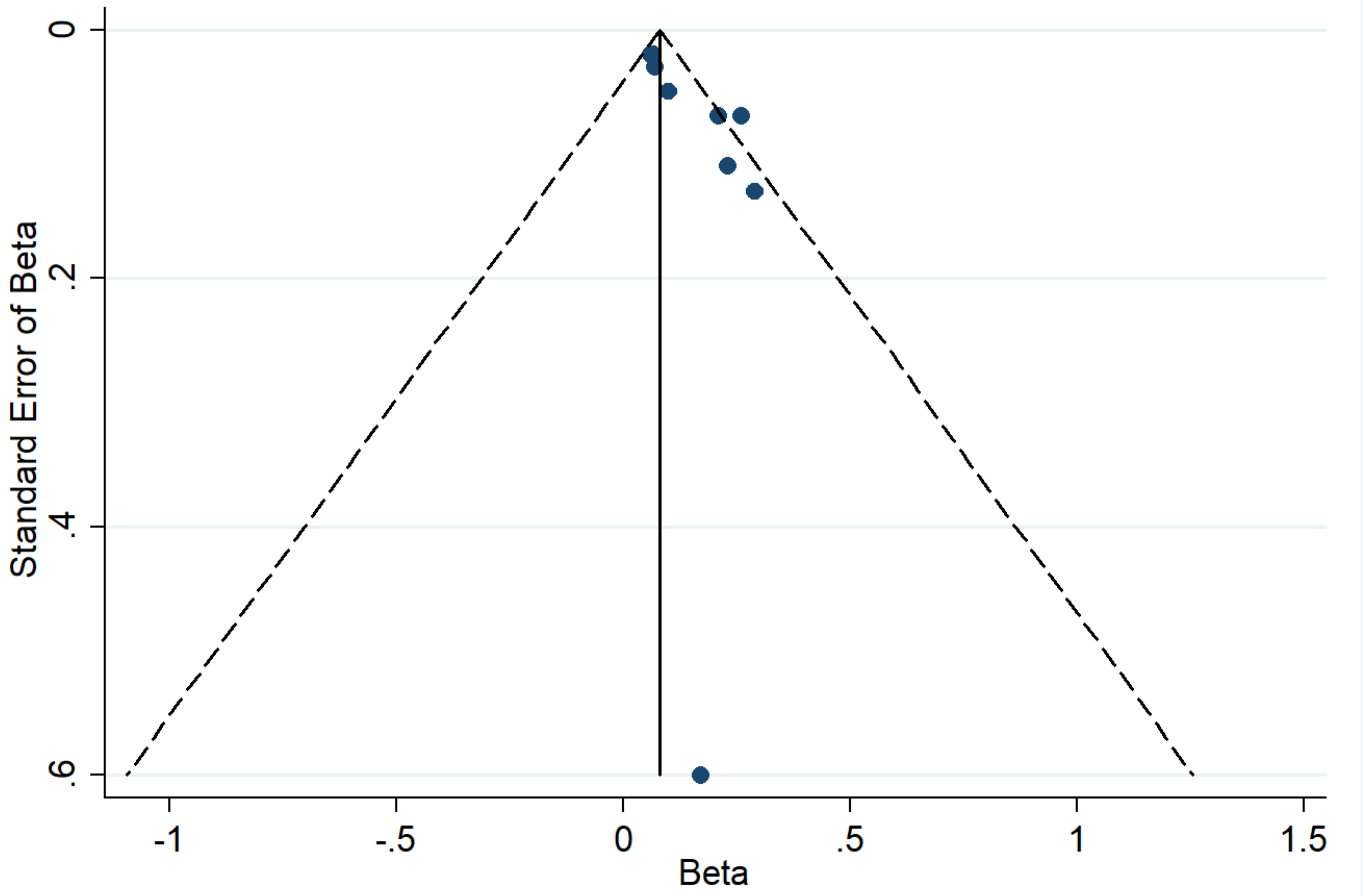

© 2021 Marino C et al. *JAMA Network Open.*

eFigure 2. Funnel Plot for Odds Ratio

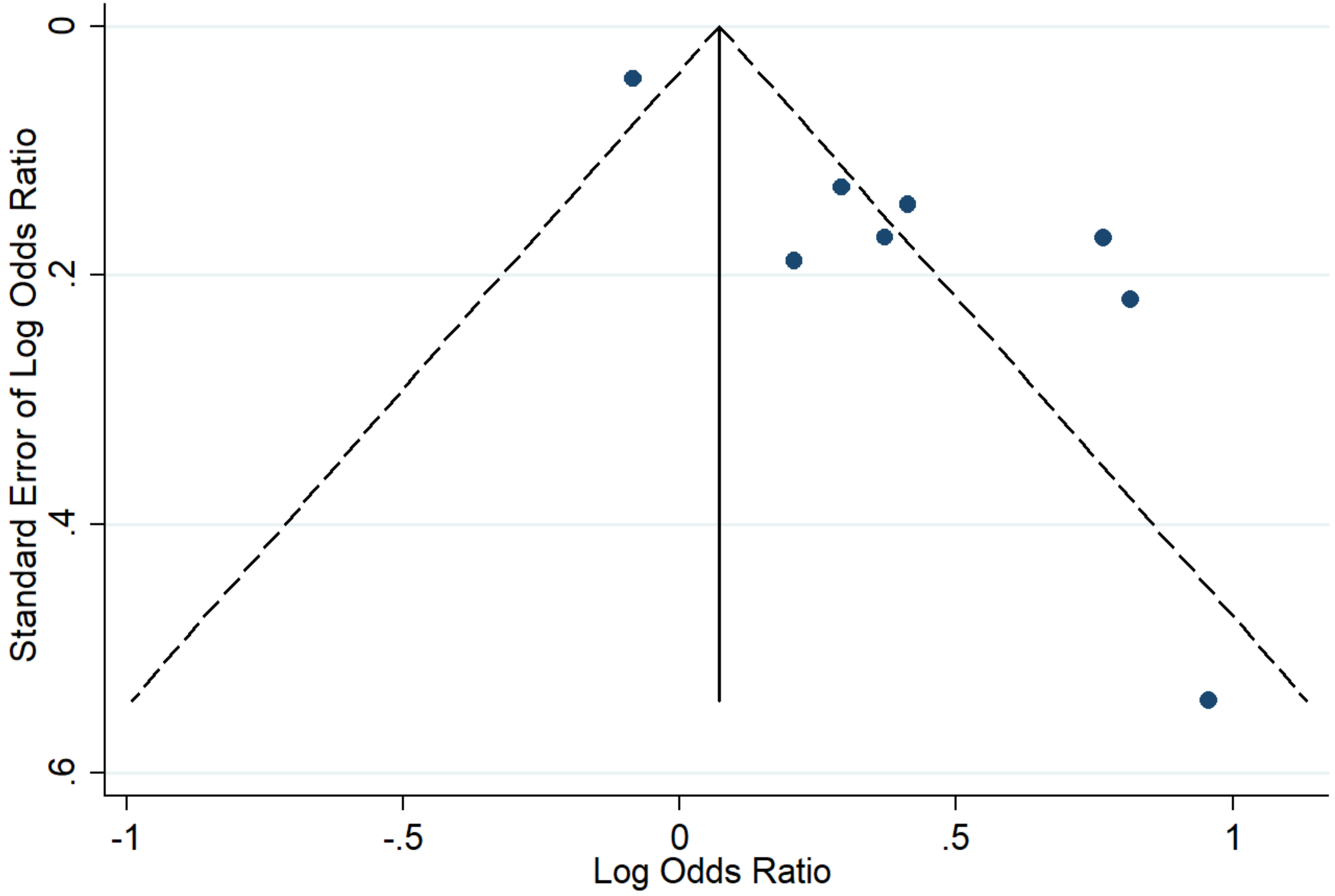

© 2021 Marino C et al. *JAMA Network Open.*

eFigure 3. Leave-One-Out Forest Plot for Standardized Beta

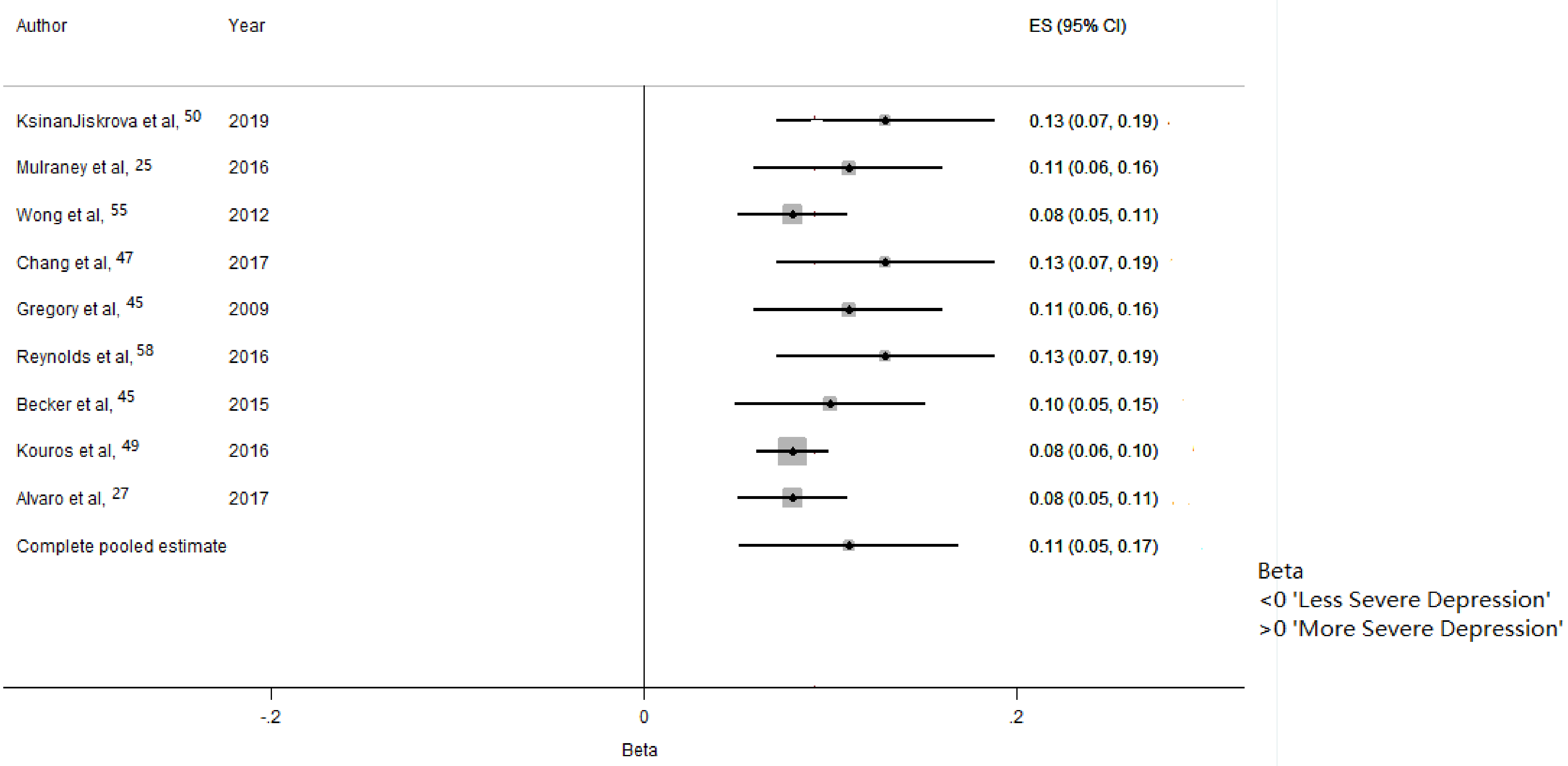

© 2021 Marino C et al. *JAMA Network Open.*

eFigure 4. Leave-One-Out Forest Plot for Odds Ratio

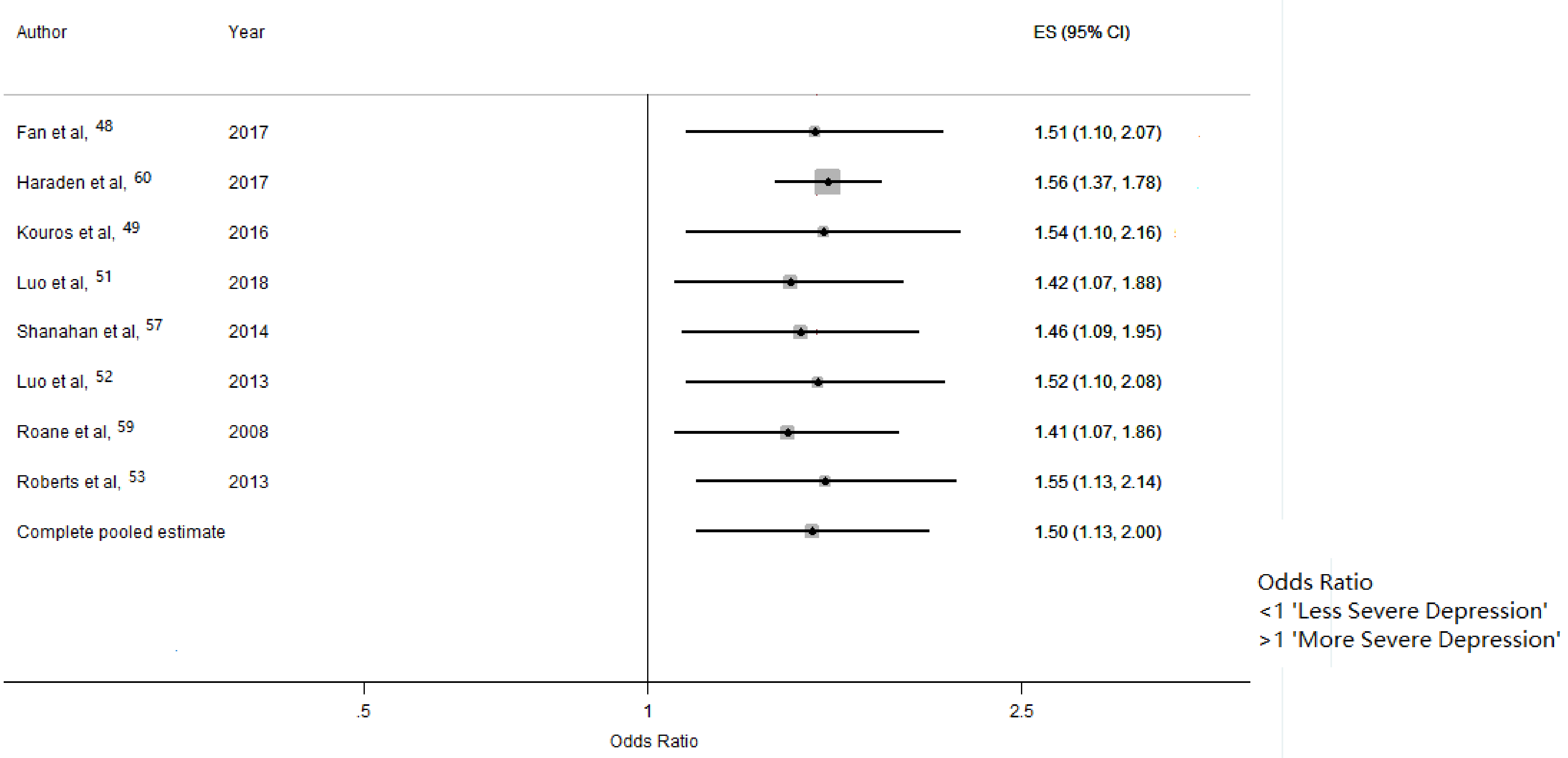

© 2021 Marino C et al. *JAMA Network Open.*

eReferences.

1. Lai YC, Huang MC, Chen HC, et al. Familiality and clinical outcomes of sleep disturbances in major depressive and bipolar disorders. *J Psychosom Res* 2014;76(1):61-7. doi: 10.1016/j.jpsychores.2013.10.020 [published Online First: 2013/12/24]
2. Rao U, Dahl RE, Ryan ND, et al. Heterogeneity in EEG sleep findings in adolescent depression: unipolar versus bipolar clinical course. *J Affect Disord* 2002;70(3):273-80. [published Online First: 2002/07/20]
3. Haddock CK, Rindskopf D., Shadish W.R. Using Odds Ratios as Effect Sizes for Meta-Analysis of Dichotomous Data: A Primer on Methods and Issues. *Psychological Methods* 1998;3:339-53.
4. Bowman NA. Effect Sizes and Statistical Methods for Meta-Analysis in Higher Education. *Research in Higher Education* 2012;53:375-82. doi: 10.1007/s11162-011-9232-5
5. Thomson HJ, Thomas S. The effect direction plot: visual display of non-standardised effects across multiple outcome domains. *Res Synth Methods* 2013;4(1):95-101. doi: 10.1002/jrsm.1060 [published Online First: 2013/06/25]
6. Johnson EO, Chilcoat HD, Breslau N. Trouble sleeping and anxiety/depression in childhood. *Psychiatry Res* 2000;94(2):93-102. [published Online First: 2000/05/16]
7. Ksinan Jiskrova G, Vazsonyi AT, Klánová J, et al. Sleep Quantity and Problems as Mediators of the Eveningness-Adjustment Link during Childhood and Adolescence. *J Youth Adolesc* 2019;48(3):620-34. doi: 10.1007/s10964-018-0965-8 [published Online First: 2018/12/04]
8. Alvaro PK, Roberts RM, Harris JK, et al. The direction of the relationship between symptoms of insomnia and psychiatric disorders in adolescents. *J Affect Disord* 2017;207:167-74. doi: 10.1016/j.jad.2016.08.032 [published Online First: 2016/10/11]
9. Becker SP, Langberg JM, Evans SW. Sleep problems predict comorbid externalizing behaviors and depression in young adolescents with attention-deficit/hyperactivity disorder. *Eur Child Adolesc Psychiatry* 2015;24(8):897-907. doi: 10.1007/s00787-014-0636-6 [published Online First: 2014/10/31]
10. Chang LY, Chang HY, Wu WC, et al. Body mass index and depressive symptoms in adolescents in Taiwan: testing mediation effects of peer victimization and sleep problems. *Int J Obes (Lond)* 2017;41(10):1510-17. doi: 10.1038/ijo.2017.111 [published Online First: 2017/05/03]
11. Doane LD, Gress-Smith JL, Breitenstein RS. Multi-method assessments of sleep over the transition to college and the associations with depression and anxiety symptoms. *J Youth Adolesc* 2015;44(2):389-404. doi: 10.1007/s10964-014-0150-7 [published Online First: 2014/07/18]
12. El-Sheikh M, Kelly RJ, Buckhalt JA, et al. Children's sleep and adjustment over time: the role of socioeconomic context. *Child Dev* 2010;81(3):870-83. doi: 10.1111/j.1467-8624.2010.01439.x
13. Fan F, Zhou Y, Liu X. Sleep Disturbance Predicts Posttraumatic Stress Disorder and Depressive Symptoms: A Cohort Study of Chinese Adolescents. *J Clin Psychiatry* 2017;78(7):882-88. doi: 10.4088/JCP.15m10206

© 2021 Marino C et al. *JAMA Network Open.*

14. Haraden DA, Mullin BC, Hankin BL. The relationship between depression and chronotype: A longitudinal assessment during childhood and adolescence. *Depress Anxiety* 2017;34(10):967-76. doi: 10.1002/da.22682 [published Online First: 2017/09/08]
15. Kouros CD, Morris MC, Garber J. Within-Person Changes in Individual Symptoms of Depression Predict Subsequent Depressive Episodes in Adolescents: a Prospective Study. *J Abnorm Child Psychol* 2016;44(3):483-94. doi: 10.1007/s10802-015-0046-3
16. Luo C, Zhang J, Chen W, et al. Course, risk factors, and mental health outcomes of excessive daytime sleepiness in rural Chinese adolescents: A one-year prospective study. *J Affect Disord* 2018;231:15-20. doi: 10.1016/j.jad.2018.01.016 [published Online First: 2018/02/01]
17. Luo C, Zhang J, Pan J. One-year course and effects of insomnia in rural Chinese adolescents. *Sleep* 2013;36(3):377-84. doi: 10.5665/sleep.2454 [published Online First: 2013/03/01]
18. Mulraney M, Giallo R, Lycett K, et al. The bidirectional relationship between sleep problems and internalizing and externalizing problems in children with ADHD: a prospective cohort study. *Sleep Med* 2016;17:45-51. doi: 10.1016/j.sleep.2015.09.019 [published Online First: 2015/10/23]
19. Reynolds KC, Alfano CA. Childhood Bedtime Problems Predict Adolescent Internalizing Symptoms Through Emotional Reactivity. *J Pediatr Psychol* 2016;41(9):971-82. doi: 10.1093/jpepsy/jsw014 [published Online First: 2016/03/27]
20. Roberts RE, Duong HT. Depression and insomnia among adolescents: a prospective perspective. *J Affect Disord* 2013;148(1):66-71. doi: 10.1016/j.jad.2012.11.049 [published Online First: 2012/12/20]
21. Schneiderman JU, Ji J, Susman EJ, et al. Longitudinal Relationship Between Mental Health Symptoms and Sleep Disturbances and Duration in Maltreated and Comparison Adolescents. *J Adolesc Health* 2018;63(1):74-80. doi: 10.1016/j.jadohealth.2018.01.011
22. Shanahan L, Copeland WE, Angold A, et al. Sleep problems predict and are predicted by generalized anxiety/depression and oppositional defiant disorder. *J Am Acad Child Adolesc Psychiatry* 2014;53(5):550-8. doi: 10.1016/j.jaac.2013.12.029 [published Online First: 2014/02/20]
23. Urrila AS, Karlsson L, Kiviruusu O, et al. Sleep complaints in adolescent depression: one year naturalistic follow-up study. *BMC Psychiatry* 2014;14:283. doi: 10.1186/s12888-014-0283-y [published Online First: 2014/10/08]
24. Wong MM, Brower KJ. The prospective relationship between sleep problems and suicidal behavior in the National Longitudinal Study of Adolescent Health. *J Psychiatr Res* 2012;46(7):953-9. doi: 10.1016/j.jpsychires.2012.04.008 [published Online First: 2012/05/01]
25. Buysse DJ, Angst J, Gamma A, et al. Prevalence, course, and comorbidity of insomnia and depression in young adults. *Sleep* 2008;31(4):473-80. doi: 10.1093/sleep/31.4.473
26. Gregory AM, Rijsdijk FV, Lau JY, et al. The direction of longitudinal associations between sleep problems and depression symptoms: a study of twins aged 8 and 10 years. *Sleep* 2009;32(2):189-99. doi: 10.1093/sleep/32.2.189 [published Online First: 2009/02/26]
27. Roane BM, Taylor DJ. Adolescent insomnia as a risk factor for early adult depression and substance abuse. *Sleep* 2008;31(10):1351-6. [published Online First: 2008/10/16]

© 2021 Marino C et al. *JAMA Network Open.*

28. Bastien CH, Vallières A, Morin CM. Validation of the Insomnia Severity Index as an outcome measure for insomnia research. *Sleep Med* 2001;2(4):297-307. doi: 10.1016/s1389-9457(00)00065-4
29. Chorpita BF, Yim L, Moffitt C, et al. Assessment of symptoms of DSM-IV anxiety and depression in children: a revised child anxiety and depression scale. *Behav Res Ther* 2000;38(8):835-55. doi: 10.1016/s0005-7967(99)00130-8
30. Achenbach TM. Manual for the ASEBA School-Age Forms & Profiles. In: Rescorla LA, ed. Burlington, VT: University of Vermont, Research Center for Children, Youth, & Families, 2001.
31. A O. Reynolds adolescent depression scale-second edition: a reliable and useful instrument. In: Gutierrez PM BC, Fang Q, Emmerich A, ed.: J Clin Psychol, 2010:1324-45.
32. Owens JA, Spirito A, McGuinn M. The Children's Sleep Habits Questionnaire (CSHQ): psychometric properties of a survey instrument for school-aged children. *Sleep* 2000;23(8):1043-51.
33. M. K. The Children's Depression, Inventory (CDI). Psychopharmacol Bull, 2001:995:98.
34. Buysse DJ, Reynolds CF, Monk TH, et al. The Pittsburgh Sleep Quality Index: a new instrument for psychiatric practice and research. *Psychiatry Res* 1989;28(2):193-213. doi: 10.1016/0165-1781(89)90047-4
35. M.E. F. Assessment of depression in childhood and adolescence: an evaluation of the Center for Epidemiological Studies Depression Scale for Children (CES-DC). In: Carey MP RL, Enyart P, Gresham F, ed.: Am J Psychiatry, 1986:1024-27.
36. Poznanski EO, Cook SC, Carroll BJ. A depression rating scale for children. *Pediatrics* 1979;64(4):442-50.
37. Keller MB, Lavori PW, Friedman B, et al. The Longitudinal Interval Follow-up Evaluation. A comprehensive method for assessing outcome in prospective longitudinal studies. *Arch Gen Psychiatry* 1987;44(6):540-8. doi: 10.1001/archpsyc.1987.01800180050009
38. Goodman R. The Strengths and Difficulties Questionnaire: a research note. *Journal of child psychology and psychiatry, and allied disciplines* 1997;38(5):581-6. doi: 10.1111/j.1469-7610.1997.tb01545.x
39. Birleson P, Hudson I, Buchanan DG, et al. Clinical evaluation of a self-rating scale for depressive disorder in childhood (Depression Self-Rating Scale). *Journal of child psychology and psychiatry, and allied disciplines* 1987;28(1):43-60. doi: 10.1111/j.1469-7610.1987.tb00651.x
40. Carskadon MA, Vieira C, Acebo C. Association between puberty and delayed phase preference. *Sleep* 1993;16(3):258-62. doi: 10.1093/sleep/16.3.258
41. Kaufman J, Birmaher B, Brent D, et al. Schedule for Affective Disorders and Schizophrenia for School-Age Children-Present and Lifetime Version (K-SADS-PL): initial reliability and validity data. *J Am Acad Child Adolesc Psychiatry* 1997;36(7):980-8. doi: 10.1097/00004583-199707000-00021 [published Online First: 1997/07/01]
42. Johns MW. A new method for measuring daytime sleepiness: the Epworth sleepiness scale. *Sleep* 1991;14(6):540-5. doi: 10.1093/sleep/14.6.540

© 2021 Marino C et al. *JAMA Network Open.*

43. BECK AT, WARD CH, MENDELSON M, et al. An inventory for measuring depression. *Arch Gen Psychiatry* 1961;4:561-71. doi: 10.1001/archpsyc.1961.01710120031004
44. Angold A, Prendergast M, Cox A, et al. The Child and Adolescent Psychiatric Assessment (CAPA). *Psychol Med* 1995;25(4):739-53. doi: 10.1017/s003329170003498x
45. Shaffer D, Fisher P, Lucas CP, et al. NIMH Diagnostic Interview Schedule for Children Version IV (NIMH DISC-IV): description, differences from previous versions, and reliability of some common diagnoses. *J Am Acad Child Adolesc Psychiatry* 2000;39(1):28-38. doi: 10.1097/00004583-200001000-00014

© 2021 Marino C et al. *JAMA Network Open.*
